# Method for In Situ On-Wafer Tensile Test of Thin Films

**DOI:** 10.3390/mi16030262

**Published:** 2025-02-26

**Authors:** Xufeng Wang, Jiakang Li, Yi Chen, Jiawei Zhou, Leijian Cheng, Dacheng Zhang

**Affiliations:** School of Integrated Circuits, Peking University, Beijing 100871, China; wangxf@pku.edu.cn (X.W.); 2301111824@stu.pku.edu.cn (J.L.); yichen@stu.pku.edu.cn (Y.C.); zhoujiawei@stu.pku.edu.cn (J.Z.); 1601111213@pku.edu.cn (L.C.)

**Keywords:** thin film, tensile test, MEMS, process quality

## Abstract

This study addresses the need for a mechanical property characterization of films during Micro-Electro-Mechanical System (MEMS) processing by proposing a novel in situ on-wafer tensile strength testing method for film materials. This method integrates the film specimen with a bulk silicon test structure during fabrication, allowing for tensile strength measurements with a resolution of 0.05 MPa using only a probe and optical microscope. Utilizing this method, we successfully performed in situ on-wafer tensile strength tests on Al films of various sizes, demonstrating the impact of the process on film mechanical properties. The results validate the potential of this structure for characterizing material mechanical properties and monitoring process quality in mass production.

## 1. Introduction

Thin films are widely used to realize various structures in MEMS and micromachined devices. In piezoresistive devices, the phenomenon of resistance change in 10–20 μm thick silicon films under stress is commonly exploited for pressure measurement [1,2]. Vertical chromium/gold thin films are crucial structures in high aspect ratio X-ray diffraction gratings [3]. In MEMS micro-mirrors, mirror thin films are used to reflect the light beam [4]. In silicon microphones, polycrystalline silicon films convert the sound pressure signal into a change in capacitance spacing, thus enabling the collection of sound signals [5].

Deposition is a commonly used method for growing thin films. During the film formation process, variations in processing conditions (such as reaction chamber environment, process parameters, deposition methods, and substrate materials) can lead to differences in composition, grain size, film thickness uniformity, surface roughness, porosity, and defects, all of which can influence the mechanical properties of the thin films. In mass production, even slight fluctuations in process conditions can cause significant changes in the mechanical properties of the films, thereby affecting the performance and reliability of the devices.

In summary, understanding the strength of thin films is crucial in the reliability design process of MEMS devices containing thin-film structures. Additionally, effective online monitoring methods for thin-film process quality are essential in MEMS mass production. Tensile testing offers several advantages, including straightforward stress distribution in specimens, ease of data processing and comparison, and sensitivity of the obtained tensile strength parameters to variations or fluctuations in process conditions. Therefore, developing a method for monitoring thin-film tensile strength in mass production holds significant importance for two reasons: (1) enabling the online monitoring of process quality to guide process improvements, and (2) providing accurate design parameters for device designers, promoting collaboration between design and manufacturing.

Conventional tensile testing of materials is typically conducted using universal testing machines, where specimen shape, testing equipment, and procedures are highly standardized [6]. After testing, the force–displacement curve is obtained, and the force at the point of specimen fracture is identified. Dividing this force by the cross-sectional area yields the tensile strength. Current tensile testing of microelectronic materials primarily focuses on materials science research, aiming to reveal microscopic mechanisms such as dislocation and grain boundary movement. Consequently, high-precision equipment such as nanoindenters [7] and the atomic force microscope (AFM) [8] and methods like nanomanipulation [9] and dielectrophoresis [10] are often employed for specimen loading, measurement, transfer, and fixation. These methods offer high measurement precision, with displacement resolution reaching the nanometer level and force resolution ranging from nanonewtons to piconewtons [11]. However, these approaches suffer from low measurement efficiency and high costs and are unsuitable for online monitoring in mass production.

Addressing the demand for the mechanical property testing of microstructures in large-scale production, R. Li et al. [12] designed an on-wafer structure that could extract the tensile strength of single-crystal silicon beams in situ using only microelectronic probes and an optical microscope. Furthermore, L. Cheng and F. Li [13,14] extended the aforementioned method to achieve a rapid in situ characterization of thin-film mechanical properties using on-wafer structures. They, respectively, designed thin-film impact-tensile structures and thin-film biaxial tension structures, but these structures lack systematic design and error analysis, have low measurement resolution (0.5 MPa), and occupy a large area (4 mm × 4 mm) on the layout, making it difficult to balance cost and parameter extraction accuracy.

In light of the aforementioned background, this paper proposes an in situ on-wafer thin-film uniaxial tensile tester for extracting the static tensile strength of freestanding thin films fabricated by the MEMS process. The structure is fabricated in the same process flow as the tested thin-film samples. The thin-film samples are loaded by the probe-pushed structure, and the real-time readings of the structure are utilized to determine the force applied to the thin film, thereby obtaining the tensile strength of the thin film. This structure can achieve a resolution of 0.05 MPa for the extraction of the tensile strength of the thin film and offers advantages such as online detection and a relatively small layout area occupation (2 mm × 4 mm) compared to previous work of the same type. It can provide process-quality monitoring for production lines and offer structural mechanical parameters for chip design.

## 2. Structure Design

**Figure 1:**
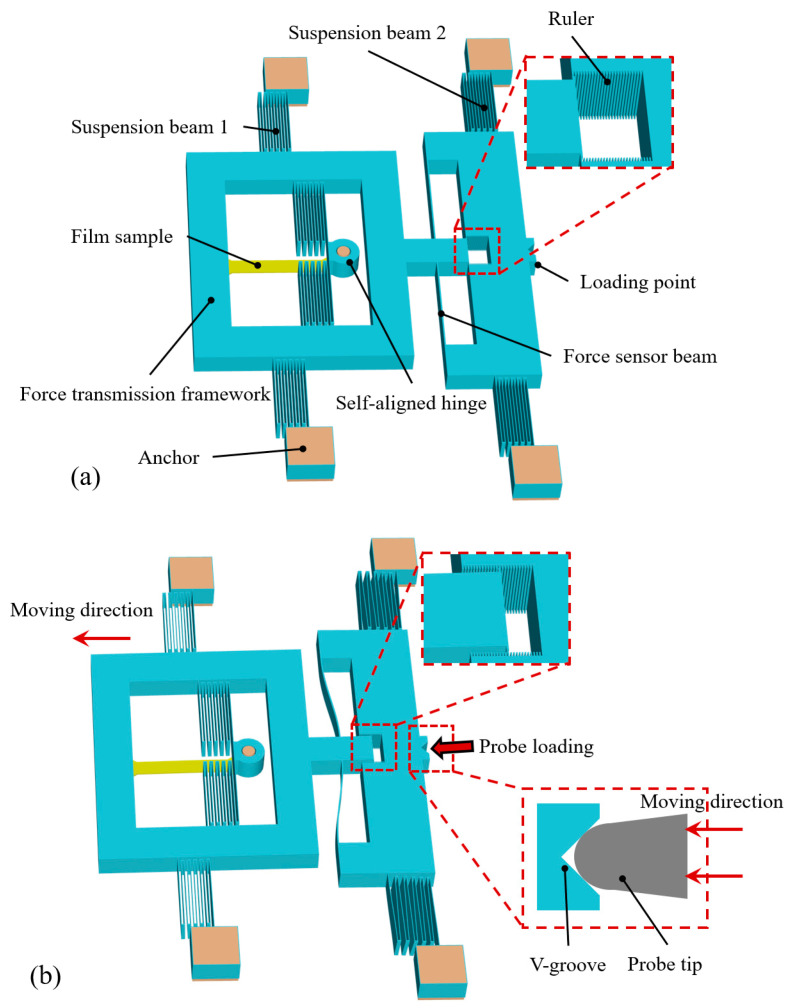
Schematic of on-wafer tester: (**a**) structure composition; (**b**) deformation during testing.

**Figure 2:**
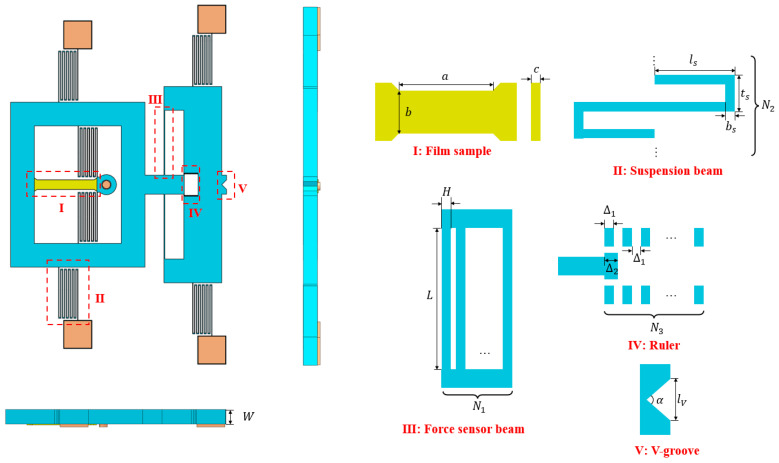
Three views of the on-wafer tester and key dimensions.

The on-wafer thin-film uniaxial tensile tester is shown in Figure 1. The on-wafer tester is mainly composed of an anchor, suspension beams, force transmission framework, self-aligned hinge, force sensor beam (FSB), ruler, and loading point. The loading point is used to receive external probe driving, and its V-groove ensures the directionality of loading. When the probe contacts the loading point and applies a load, the on-wafer tester moves, and the thin-film sample undergoes uniaxial tensile deformation under the pulling of the force transmission framework and the constraint of the self-aligned hinge, as shown in Figure 1b. The readings of the ruler provide data about the overall deflection value, which can be subsequently used in the measurement model to determine the force on the thin film, further allowing for the calculation of its strength. Figure 2 shows three views of the structure and a dimensional representation of the key modules.

### 2.1. Mechanical-Lumped Model

**Figure 3:**
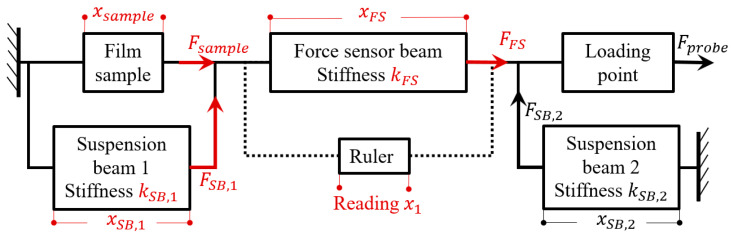
Mechanical-lumped model of on-wafer tester.

The force application of the structure under quasi-static conditions is modeled using an equivalent circuit approach. The rigid blocks are equivalent to wires, forces are equivalent to currents, displacements are equivalent to voltages, and anchor points are equivalent to ground connections. The mechanical-lumped model of the on-wafer tester is shown in Figure 3.

The mechanical-lumped model of the on-wafer tester can also be described by the following equations.

(1) $$F_{FS}=F_{sample}+F_{SB,1}$$

(2) $$x_{sample}=x_{SB,1}$$

(3) $$x_{FS}=x_{1}$$

(4) $$F_{FS}=k_{FS}x_{FS}$$

(5) $$F_{SB,1}=k_{SB,1}x_{SB,1}$$

(6) $$F_{sample}=k_{sample}x_{sample}$$

Among those, $F_{FS}$, $F_{sample}$, and $F_{SB,1}$ represent the forces on the force sensor beam, the film sample, and suspension beam 1, respectively. $x_{FS}$, $x_{sample}$, and $x_{SB,1}$ represent the deformations of the force sensor beam, the film sample, and suspension bean 1, respectively. $k_{FS}$, $k_{sample}$, and $k_{SB,1}$ represent the stiffnesses of the force sensor beam, the film sample, and suspension beam 1, respectively. $x_{1}$ represents the deflection reading of the ruler. As can be seen from the above equation,

(7) $$F_{sample}=k_{FS}x_{1}-F_{SB,1}$$

Ensure that $k_{SB,1}\ll k_{sample}$ when designing, then the force on the sample can be approximated as follows:

(8) $$F_{sample}=k_{FS}x_{1}$$

### 2.2. Model of Force Sensor Beam

**Figure 4:**
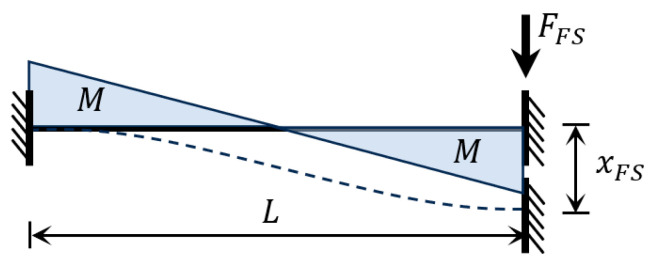
Deformation and bending moment of load sensor beam under loading.

The force sensor beam is designed to convert the force exerted on the film into the deformation of the beam, which is more convenient for measurement. The displacement difference $x_{1}$ at the two ends of the force sensor beam during film loading is measured, and this value is then substituted into Equation (8) to calculate the force exerted on the film. Due to the symmetric structure, only half of the force sensor beam is analyzed for force distribution, simplifying the force model to a guide beam, as shown in Figure 4.

The equivalent elastic coefficient under the illustrated load is calculated by structural mechanics:

(9) $$k_{FS}=\frac{F_{FS}}{x_{FS}}=\frac{12EI}{L^{3}}=\frac{EWH^{3}}{L^{3}}$$

where E is the Young’s modulus of the beam, I is the moment of inertia of the section, L, H, and W are the length, width, and thickness of the beam, respectively.

The stress in the guide beam is analyzed. The section experiencing the maximum bending moment is located at the ends of the beam, and the maximum bending moment is given by

(10) $$M_{max}=\frac{F_{FS}L}{2}$$

The maximum tensile stress resulting from the bending moment occurs on the outer side of the section, and the maximum stress value is given by

(11) $$\sigma_{1}=\frac{M_{max}H}{2I}=\frac{F_{FS}LH}{4I}$$

There are axial constraints at the ends of the beam; therefore, it is elongated during actual deformation, and the effect of axial tension-induced stress must be considered. According to the principle of displacement superposition, the axial elongation caused by the axial force is given by

(12) $$\delta (x)=\frac{{F_{FS}}^{2}L^{4}}{240E^{2}I^{2}}x$$

where x is the coordinate along the beam axis. Therefore, the normal stress caused by axis elongation is as follows:

(13) $$\sigma_{2}=\frac{E\delta (x)}{x}=\frac{{F_{FS}}^{2}L^{4}}{240EI^{2}}$$

Combined with Formula (11), the maximum normal stress on the guide beam can be obtained as follows:

(14) $$\sigma_{max}=\sigma_{1}+\sigma_{2}=\frac{F_{FS}LH}{4I}+\frac{{F_{FS}}^{2}L^{4}}{240EI^{2}}$$

Based on this, the fracture reliability of the force sensor beam can be designed such that the maximum stress calculated using the above equation should not exceed the critical fracture stress.

### 2.3. Model of Suspension Beam

The suspension beam is used to connect the on-wafer tester structure and anchor points, which can hold the on-wafer tester and guide its movement in the desired direction. The design principle of the spring is: as follows the stiffness along the direction of structure movement $k_{s}$ is as small as possible, the stiffness outside the structure plane $k_{z}$ is as large as possible, and the spring does not break within the range of motion of the suspension structure, that is, the maximum first principal stress is less than the breaking strength.

**Figure 5:**
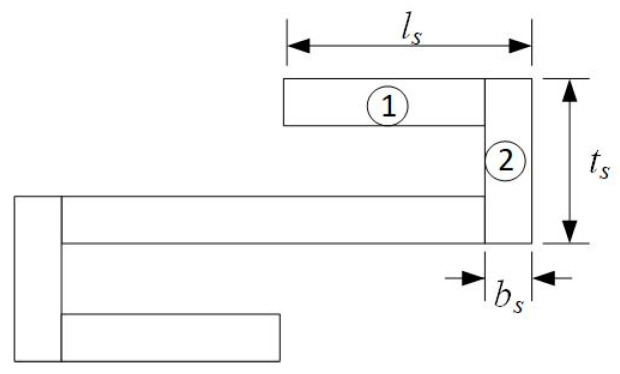
Schematic of a unit of L-shaped spring.

Here, the L-shaped spring is selected as the suspension beam. The L-shaped spring can be regarded as consisting of multiple units, as shown in Figure 5, repeatedly connected.

According to the research of H. Li et al. [15], the stiffness coefficient of the L-shaped spring in the movement direction of tester is as follows:

(15) $$k_{s}=\frac{E{b_{s}}^{3}W}{n(16l_{s}^{3}+24l_{s}^{2}t_{s}+2b_{s}^{2}t_{s})}$$

where $l_{s}$ is the length of the spring unit, $b_{s}$ is the line width of the spring unit, $t_{s}$ is the spacing of the spring, and n is the number of units.

The stress of the spring during deformation is analyzed, and the single spring unit is selected for force analysis. Due to the symmetry of the spring, the force of the part labeled 1 is consistent with that of other parts with the same shape. Similarly, the part labeled 2 is also consistent with that of the part with the same shape, so only 1 and 2 parts are analyzed. According to the mechanics of materials, it can be calculated that the first part is only affected by the bending moment, and the maximum should be as follows:

(16) $$\sigma =\frac{6F_{SB}l_{s}}{{b_{s}}^{2}W}$$

The second part is subjected to both bending and tensile stress, the maximum stress is as follows:

(17) $$\sigma =\frac{6F_{SB}(l_{s}+\frac{b_{s}}{2})}{{b_{s}}^{2}W}+\frac{F_{SB}}{b_{s}W}$$

It can be calculated from the two formulas that the stress concentration point is at the right corner of the spring. According to the experimental results of R. Li, et al. [12], the dimensional design can be carried out according to the critical fracture stress of 1 GPa, and the dimensional parameters of the spring can be finally determined.

### 2.4. Fixation of Film Sample

**Figure 6:**
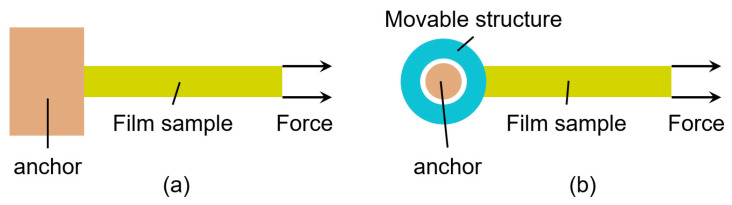
(**a**) Fixed constraint; (**b**) hinged support.

According to the width of film sample, the film is fixed in two forms: fixed constraint and hinged support, as shown in Figure 6.

**Figure 7:**
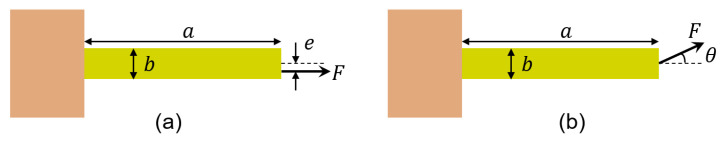
(**a**) Displacement deviation; (**b**) angular deviation.

When applying load to a film specimen, it is often challenging to ensure the exact direction of the load, leading to force deviations, which can be categorized into displacement deviation and angular deviation, as shown in Figure 7. Displacement deviation refers to the single-axis tensile force acting away from the centerline of the film, which manifests as an inaccurate position of the probe during loading. Angular deviation refers to the single-axis tensile force direction deviating from the centerline of the film by a certain angle, which is reflected in the probe’s motion direction deviating from the axis.

For a film with a length a, width b, and thickness c that is a fixed constraint at one end and subjected to uniaxial tensile force $F_{sample}$, if there are no loading deviations, the normal stress experienced by the film is given by

(18) $$\sigma_{0}=\frac{F_{sample}}{bc}$$

When there is a loading displacement deviation of size e, as shown in Figure 7a, the maximum normal stress experienced by the film is

(19) $$\sigma_{max,1}=\frac{F_{sample}}{bc}+\frac{6F_{sample}e}{cb^{2}}$$

The resulting strength error is

(20) $$\eta_{1}=1-\frac{\sigma_{0}}{\sigma_{max,1}}=\frac{6e}{6e+b}$$

When there is an angle loading deviation of θ, as shown in Figure 7b, the maximum normal stress experienced by the film is

(21) $$\sigma_{max,2}=\frac{F_{sample}\cos\theta }{bc}+\frac{6F_{sample}\sin\theta a}{cb^{2}}$$

The resulting strength error is

(22) $$\eta_{2}=1-\frac{\sigma_{0}}{\sigma_{max,2}}=1-\frac{1}{\cos\theta +\frac{6a}{b}\sin\theta }$$

According to Equation (22), when the film width b is large, the effects of both loading deviations on the final results are small. Therefore, for films with a large width, it is appropriate to use fixed constraints directly. For films with a smaller width, self-alignment hinge structures can be used to constrain the film, achieving adaptive control of the loading direction and position to eliminate the effects of loading deviations on the measurement results.

## 3. Fabrication

### 3.1. Process Flow

**Figure 8:**
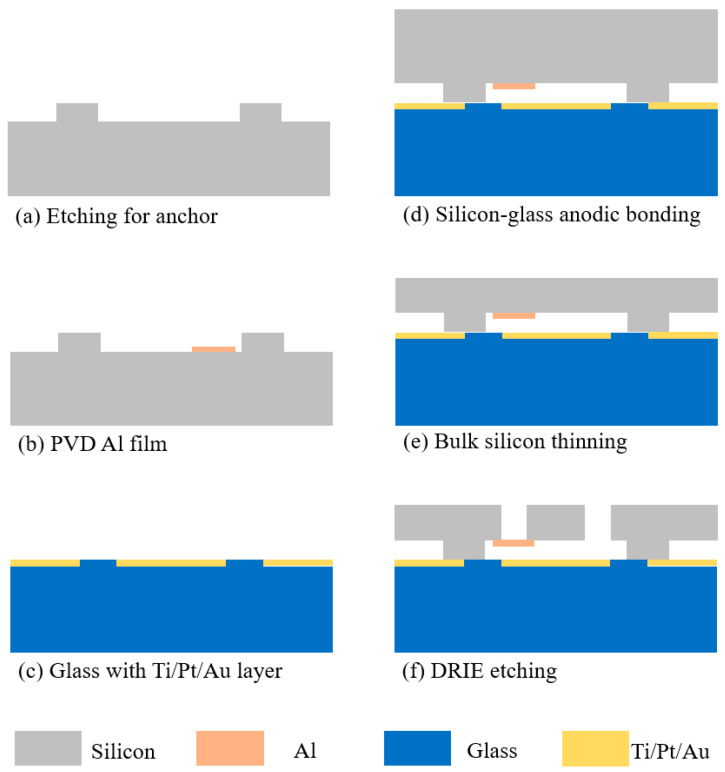
Fabrication process flow, the silicon serves as the top wafer, while the glass functions as the bottom wafer.

Silicon on glass (SOG) is a commonly used process flow for preparing bulk silicon movable MEMS structures. This process enables the fabrication of movable silicon structures on glass substrates. The tester described in this paper can be prepared by adding the deposition and release of thin film on the basis of the process flow, shown in Figure 8. We used n-type (0.001–0.003 Ω cm) (100) single-crystal silicon wafers (4 inches, 390 ± 10 μm width) as the structural material. The anchors with a thickness of 5 μm were formed by deep reactive ion etching (DRIE) with masking material of photoresist, which was removed after etching. No oxide etching is required after anchor point formation. The width of the anchor point is usually more than 100 μm to ensure bond quality. Then, the aluminum film with 99.99% purity is sputtered (1000 W, 4–4.7 × 10^−4^ Pa, Ar Flow = 15 sccm) and patterned (Wet etch, photoresist mask) on the step under the anchor point and graphically used as test sample (thickness shown in Table 1) with no subsequent treatment.

Meanwhile, Ti/Pt/Au (300/400/2200 Å) patterns were fabricated on glass (Pyrex 7740, Valley Design, Shirley, MA, USA) using a lift-off process to prevent footing effect in DRIE. After anodic bonding (340 °C, 800 V, 4 mA, 260 N) of silicon and glass, the silicon wafer was etched to the designed thickness (60 μm) with KOH solution (80 °C, 320–340 min). An aluminum thin film (1000 Å) is deposited on the top surface of the silicon wafer using PVD and patterned (Wet etch, photoresist mask) to serve as a mask for the subsequent DRIE process for structural release. Finally, movable suspension structures were formed by DRIE.

**Figure 9:**
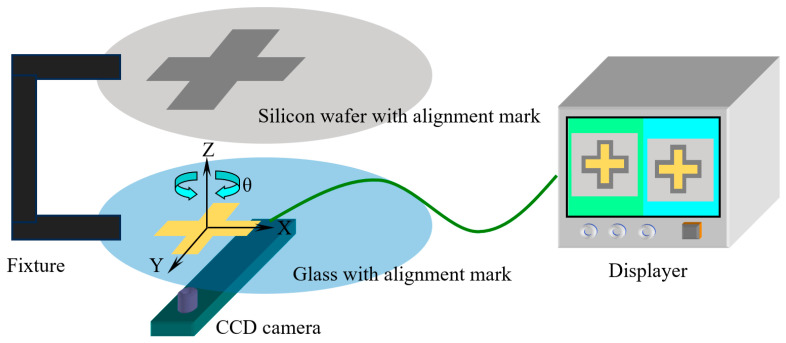
Schematic diagram of silicon-glass bonding alignment.

It is important to note that the alignment of silicon-glass bonding is achieved using double-sided lithography alignment technology, as illustrated in Figure 9. The specific process is as follows:

1. Predefined alignment mark patterns are created on both the silicon wafer and the glass during the steps of silicon anchor point etching (Figure 8a) and metal deposition on the glass (Figure 8c), respectively.
2. The bonding surfaces of the glass and silicon wafer are positioned opposite each other and secured in a specialized alignment fixture for bonding. The fixture is then placed on the lithography machine, with the silicon wafer on top and the glass substrate underneath.
3. A CCD camera located beneath the lithography machine’s stage captures the alignment marks on the silicon wafer through the glass from below and records their images on the display.
4. The CCD camera automatically adjusts its focus to the glass substrate. By fine-tuning the *x*-axis, *y*-axis, and angular θ knobs, the alignment marks on the glass are aligned with those of the silicon wafer recorded on the display.
5. The fixture locks the relative positions of the two substrates. The fixture is then removed from the lithography machine and transferred to the bonding machine to complete the bonding process.

The bonding accuracy is ±2 μm.

### 3.2. Process Error Analysis

**Figure 10:**
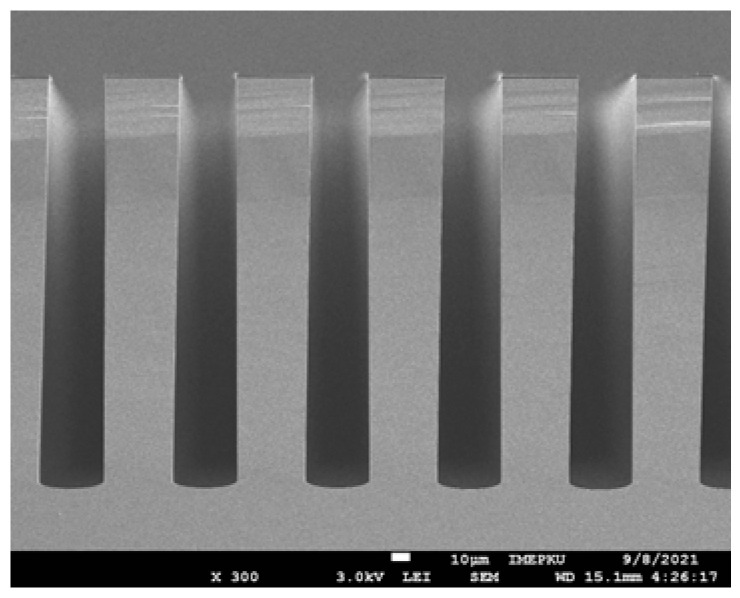
Sidewall tapering of high-aspect-ratio trench fabricated by DRIE.

During the etching process of the tester structure, inevitable line width loss and sidewall tapering occur, leading to deviations in the structural geometry from the designed specifications and changes in mechanical properties. Figure 10 illustrates the typical morphology of deep trenches etched using DRIE.

**Figure 11:**
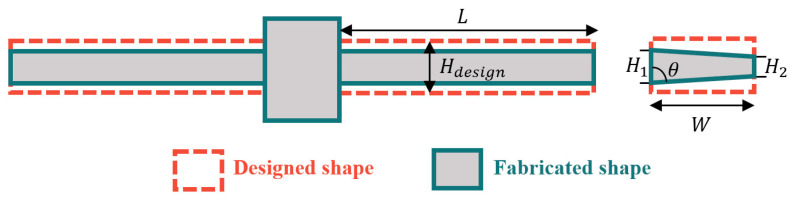
Geometrical deviation of force sensor beam due to DRIE.

Among the various modules of the test machine, the stiffness values of the force sensor beam are most affected by these non-ideal effects. As shown in Figure 11, due to the line width loss and sidewall tapering during etching, the cross-sectional shape of the force beam may deviate from the designed rectangular shape and become approximately trapezoidal in appearance.

The deviation of the section geometry from the design value can be described by the upper line width loss $∆H_{1}$ and the lower line width loss $∆H_{2}$:

(23) $$∆H_{1}=\frac{H_{design}-H_{1}}{2}$$

(24) $$∆H_{2}=\frac{H_{design}-H_{2}}{2}$$

where $H_{design}$ is the design line width of the force measuring beam, and $H_{1}$ and $H_{2}$ are the two bottom lengths of the trapezoid of the section, then the sidewall inclination angle is as follows:

(25) $$\theta =\arctan\frac{∆H_{2}-∆H_{1}}{W}$$

At this time, the moment of inertia of the section of force sensor beam is

(26) $$I=\frac{W}{48}\left(H_{1}^{2}+H_{2}^{2}\right)\left(H_{1}+H_{2}\right)$$

The resulting measurement intensity error is

(27) $$\eta =1-\frac{\sigma }{\sigma_{0}}=1-\frac{k_{FS}}{k_{FS,0}}=1-\frac{I}{I_{0}}$$

## 4. Experimental Results and Discussion

Ten film samples of different sizes and corresponding tester structures of different sizes were prepared, as shown in Table 1. The other dimensions of on-wafer tester are independent of dimensions of the film samples, as shown in Table 2.

**Figure 12:**
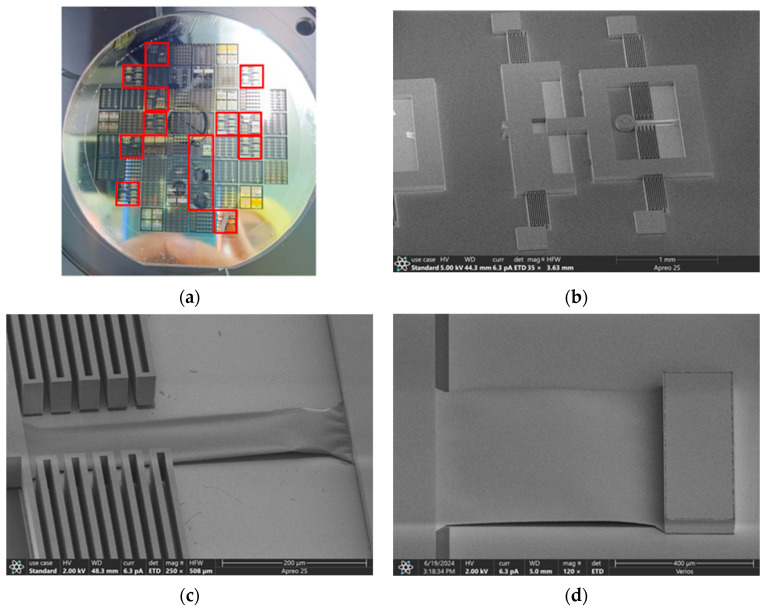
(**a**) Fabricated wafer and tested cells; SEM photos of (**b**) the whole picture of tester; (**c**) narrow film sample; (**d**) wide film sample.

Figure 12a shows the fabricated wafer, with the red box corresponding to the test unit. Figure 12b shows the overall picture of the tester captured by SEM. Figure 12c,d show the aluminum film sample with different widths.

Since the strength measurement is obtained by determining the force applied to the thin-film specimen through deformation monitoring of a force-sensor beam with known stiffness $k_{FS}$, followed by division through the cross-sectional area bc of the film, the theoretical measurement resolution ${∆}_{\sigma }$ can be calculated using the following expression:

(28) $${∆}_{\sigma }=\frac{{∆}_{F}}{bc}=\frac{k_{FS}\delta }{bc}=\frac{EWH^{3}\delta }{L^{3}bc}$$

where ${∆}_{F}$ represents the theoretical force resolution, and δ denotes the displacement resolution in optical measurement. In our experimental configuration, an optical microscope integrated with a sub-pixel recognition algorithm [16] was employed for displacement measurement, achieving a displacement resolution δ of 0.01 μm. By substituting the above data and the data in Table 1 into Equation (28), the theoretical measurement resolution can be obtained as 0.05 MPa.

**Figure 13:**
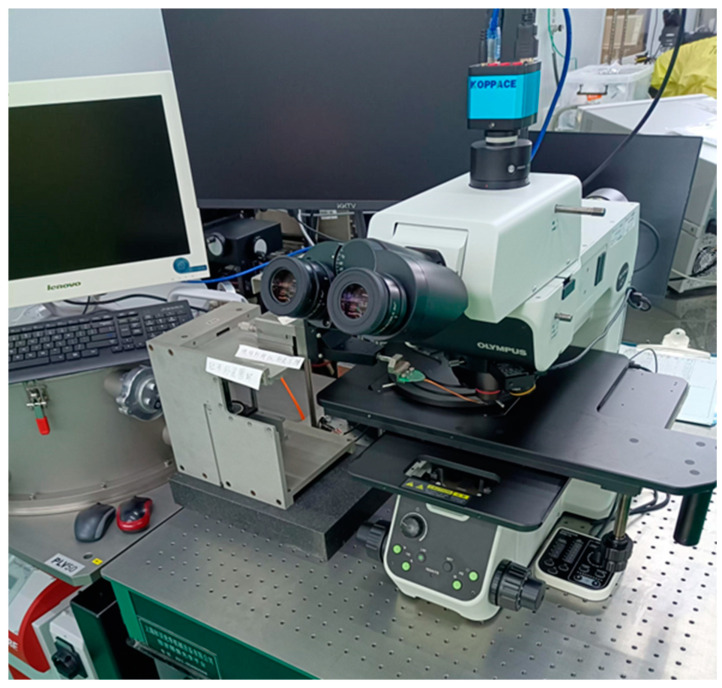
Testing equipment consisting of Olympus MX63 optical microscope, custom-designed probe holder, and video recording system.

The ultimate tensile strength of ten films of different sizes was tested using this tester. The testing equipment consists of an Olympus MX63 (Tokyo, Japan) optical microscope, a custom-designed probe holder, and a video recording system, shown in Figure 13. The detailed testing procedure is described as follows:

1. Place the wafer to be tested on the microscope stage and level it.
2. Adjust the probe so that the probe tip is positioned at the V-groove of the probe loading point and begin video recording. Move the probe in the direction indicated in Figure 1 to drive the testing structure to apply an increasing axial tensile force to the thin film.
3. Observe the deformation of the thin-film test structure in real-time through the microscope. Once the thin-film test structure fractures, stop the driving load and cease video recording.
4. Review the video recording to read the ruler reading $x_{1}$ at the moment of fracture of the thin film.
5. Substitute $x_{1}$ into the theoretical model from the previous section to calculate the tensile strength of the thin film.

**Figure 14:**
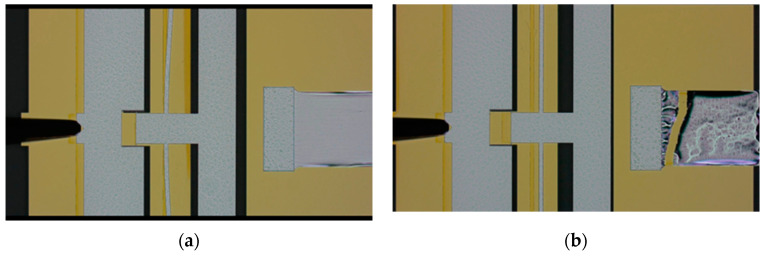
Film samples during testing: (**a**) plastic deformation; (**b**) fracture.

**Figure 15:**
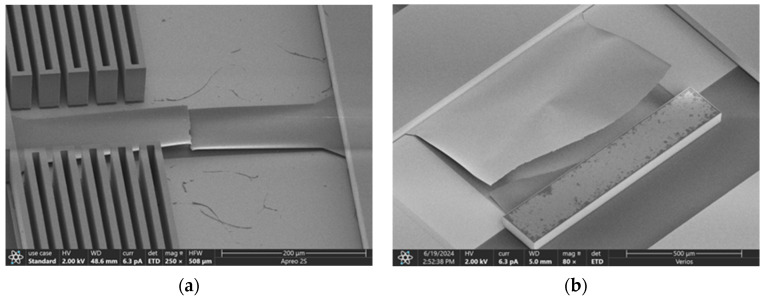
SEM photos of fractured film: (**a**) narrow film; (**b**) wide film.

**Figure 16:**
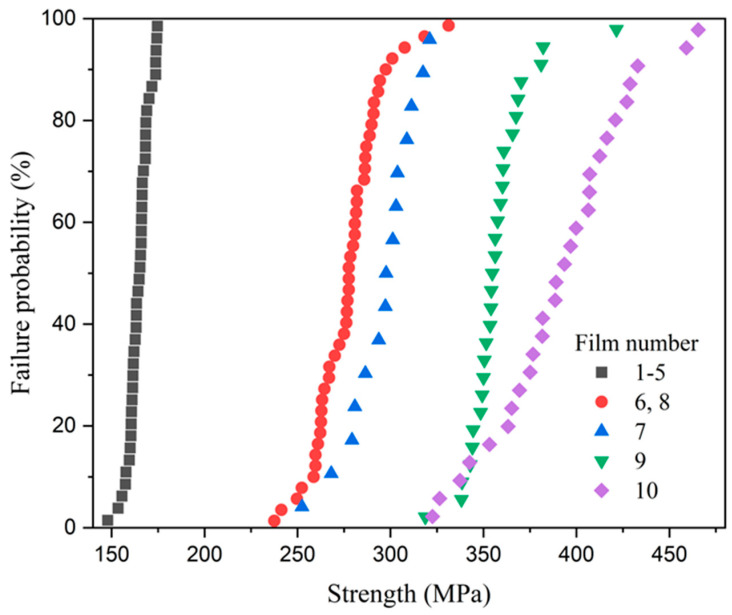
Failure probability distribution of films of different dimensions.

Figure 14 shows the process of plastic deformation and fracture of the film sample during the test. Figure 15 shows the SEM photos of films with different widths after fracture. Grouped measurements were performed on multiple thin-film specimens exhibiting distinct geometric dimensions as specified in Table 1. The tested strength results were grouped into categories with similar values, and the failure probabilities of the films with different sizes are shown in Figure 16. Each data point corresponds to the mechanical strength measurement of an individual thin-film specimen.

Based on the test results, the following conclusions can be drawn:

1. No correlation between strength and position on the wafer was observed.
2. The analysis of the strength results from Groups 1–5 reveals that the ultimate strength is independent of the film length, indicating no size effect on brittle fracture. This can be attributed to the fact that new defects initiate during the stretching process before old defects propagate, with the fracture process dominated by ductile fracture.
3. Samples from Groups 1–5 exhibited immediate fracture after the elastic stage, with no distinct yield phenomenon observed. In contrast, Groups 6, 7, and 8 showed a relatively short yield stage followed by immediate fracture, with the ultimate strength approximately equal to the yield strength. Groups 9 and 10 demonstrated a more pronounced yield phenomenon, characterized by (1) a significant elongation of the aluminum film specimen, as shown in Figure 14a; (2) the ruler reading remaining relatively constant during the test, with no change in response to probe movement.
4. The fracture/yield strength of the samples increased with increasing cross-sectional area. This is attributed to the following reasons: (1) wider aluminum films provide more freedom for dislocation generation and movement, exhibiting better plasticity; (2) thinner aluminum films are more affected by surface oxide layers, leading to localized stress concentration and thus a decrease in yield strength.

Table 3 provides a comparison between the aluminum thin-film strength measured in this study and previously reported results. It can be observed that the strength of macroscopic bulk materials, micrometer-thick films, and nanometer-thick films generally increases in that order. However, as noted in Conclusion 4 above, the results of this study indicate that the strength of 500 nm thick films is higher than that of 100 nm thick films, which may be attributed to the influence of the natural oxide layer. This raises two important questions: (1) Under the condition of the natural oxide layer’s presence, does there exist an optimal film thickness that maximizes strength? (2) How can the intrinsic properties of nanometer-thick materials be measured while minimizing the impact of the natural oxide layer? These questions warrant further in-depth investigation.

## 5. Conclusions

Films are critical structures in MEMS, and their mechanical properties are strongly influenced by the fabrication process. This study proposes an in situ on-wafer testing method for characterizing the mechanical properties of films fabricated during MEMS processing. This method involves the fabrication of a test structure using conventional bulk silicon MEMS processing techniques and integrating it with the film specimen. By driving the test structure with a probe and recording the readings when the film fails, the tensile strength of the film is calculated using a mechanical model. The measurement principles, design methods, and error analyses of the test structure are discussed. Using this method, we successfully extracted the tensile strengths of films of various sizes and investigated the relationships between strength differences and process parameters and dimensions. In theory, this method is applicable to metals such as aluminum and gold. With slight adjustments to the process flow, it can also be extended to the testing of non-metallic thin films, such as silicon oxide and polysilicon. This method offers the advantages of high efficiency and ease of use, with the measurement resolution meeting the requirements for process quality control. It holds potential for mechanical property monitoring during mass production.

## Figures and Tables

**Table 1:** Film sample and corresponding tester structure dimensions.

No.	Al Film Sample			Force Sensor Beam			Constraint
	a (μm)	b (μm)	c (nm)	H (μm)	L (μm)	$N_{1}$	
1	200	100	100	10	685	1	Hinge
2	400	100	100	10	685	1	Hinge
3	600	100	100	10	685	1	Hinge
4	800	100	100	10	685	1	Hinge
5	1000	100	100	10	685	1	Hinge
6	600	500	100	28	1170	1	Fixed
7	600	1000	100	28	1170	2	Fixed
8	600	100	500	28	1170	1	Fixed
9	600	500	500	28	1170	5	Fixed
10	600	1000	500	28	1170	10	Fixed

**Table 2:** Other common on-wafer tester dimensions.

**Suspension Beam**							**Structure Thickness**	
$l_{s}$ **(μm)**	$t_{s}$ **(μm)**		$b_{s}$ **(μm)**		$N_{2}$		W **(μm)**	
520	30		10		5		60	
**Ruler**						**V-groove**		
${∆}_{1}$ **(μm)**		${∆}_{2}$ **(μm)**		$N_{3}$		α **(°)**		$l_{V}$ **(μm)**
3		5		25		90		100

**Table 3:** Summary of tensile strength of aluminum.

Reference	This Research	Cheng [17]		Read et al. [18]	Smithells [19]		
**Thickness**	100–500 nm	100 nm		0.2–5 μm	Bulk		
**Purity**	99.99%	99.99%		99.999%	99.99%		
**Preparation and processing**	Sputtering	Sputtering	Evaporation	E-beam evaporation	O	H4	H8
**Strength (MPa)**	156–465	450–490	325–390	124–176	55	85	110

## Data Availability

The original contributions presented in this study are included in the article. Further inquiries can be directed to the corresponding author(s).
